# Pentavalent Vanadium Species as Potential Corrosion Inhibitors of Al_2_Cu Intermetallic Phase in the Sulfuric(VI) Acid Solutions

**DOI:** 10.3390/ma13081946

**Published:** 2020-04-21

**Authors:** Przemysław Kwolek, Barbara Kościelniak, Magdalena Wytrwal-Sarna

**Affiliations:** 1 Department of Materials Science, Faculty of Mechanical Engineering and Aeronautics, Rzeszow University of Technology, Żwirki i Wigury 4, 35-036 Rzeszów, Poland; b.koscielnia@prz.edu.pl; 2 Academic Centre for Materials and Nanotechnology, AGH University of Science and Technology, Kawiory 30, 30-055 Kraków, Poland; wytrwal@agh.edu.pl

**Keywords:** intermetallics, selective corrosion, sodium orthovanadate, isopolyoxoanions

## Abstract

The objective of this work was to test vanadium isopolyoxoanions as potential corrosion inhibitors of the intermetallic phase Al_2_Cu in sulfuric acid solutions at pH = 1.3 and 2.5. The intermetallic was melted in an electric arc furnace. Its phase composition was confirmed using X-ray diffraction, light microscopy, and differential scanning calorimetry. Then Al_2_Cu corrosion kinetics was studied. Chemical composition of the solution after corrosion was determined using inductively coupled plasma-optical emission spectroscopy. The surface of corroded specimens was analyzed using scanning electron microscopy and X-ray photoelectron spectroscopy. Subsequent electrochemical studies involved determination of open-circuit potential, electrochemical impedance spectra, and polarization curves. It was found that the Al_2_Cu phase corrodes selectively and vanadium isopolyoxoanions increase this process both at pH = 1.3 and 2.5 with two exceptions. Corrosion inhibition was observed for 100 and 200 mM of Na_3_VO_4_ at pH 1.3, with inhibition efficiency 78% and 62% respectively, due to precipitation of V_2_O_5_.

## 1. Introduction

Aluminum is the most widely applied non-ferrous metal. Components made of aluminum alloys are mainly used in transportation, engineering, construction, and packaging, where their good strength, corrosion, and wear resistance is often required. Phase composition of aluminum alloys can be very complex because this metal forms numerous intermetallics with alloying elements and impurities. These intermetallics significantly affect alloy properties e.g., strength and corrosion resistance. One of the most important intermetallic in aluminum alloys is Al_2_Cu. It ensures good strength of Al-Cu-Mg-Mn and Al-Zn-Mg-Cu wrought alloys as well as Al-Cu cast alloys [1]. Unfortunately, the precipitation of intermetallic in the matrix diminishes corrosion resistance of these alloys. This occurs due to strong galvanic coupling between Al_2_Cu and the matrix and local dissolution of the latter [2,3,4,5,6,7,8,9,10]. At the same time, Al_2_Cu itself is susceptible to the selective corrosion in acidic and alkaline solutions [2,3,11,12].

Corrosion studies of intermetallic phases are important for understanding the corrosion mechanism of copper-rich aluminum alloys. Components made of these alloys are usually applied in neutral environments. Their surface treatment, however, often requires acid etching. This occurs e.g., when anodic coatings are stripped in hot orthophosphoric acid solution. Presently, industry commonly uses chromium trioxide to prevent corrosion of the metallic substrate during etching/stripping [13]. Unfortunately, hexavalent chromium species are highly toxic [14]. Thus, new, less toxic inhibitors must be developed. Sodium molybdate was successfully applied instead of chromium trioxide in the stripping bath. Unfortunately, sufficient corrosion inhibition was achieved only for aluminum 1050, but not for commonly applied 2024 alloy [15,16,17]. It was also found that sodium molybdate decreases the dissolution rate of anodic coating [18].

Recently, sodium orthovanadate, tungstate, and molybdate, were tested as the potential inhibitors of corrosion of Al_2_Cu intermetallic phase in orthophosphoric acid [12]. When adding increasing amounts of a mineral acid to aqueous solutions of orthometalates, addition and condensation processes occur and isopolyoxoanions are formed. They are composed of a transition metal, oxygen, and hydrogen. Condensation in a solution containing e.g., orthophosphoric or silicic acid gives heteropolyoxoanions. They incorporate one or more elements e.g., phosphorus or silicon, in addition to the transition metal, oxygen and hydrogen [19]. It was shown that neither molybdenum nor tungsten heteropolyoxoanions inhibit corrosion of intermetallic Al_2_Cu [12]. Corrosion was inhibited however in 0.5 and 1 M H_3_PO_4_ due to reduction of vanadium heteropolyoxoanions and precipitation of an insoluble salt [12,20].

In this work, isopolyoxovanadates were tested as corrosion inhibitors of intermetallic Al_2_Cu. Literature survey indicated that tetrahedrally coordinated species, existing in mildly acidic solutions, such as [H_2_VO_4_]^−^ and [V_4_O_12_]^4−^, inhibit the cathodic process on aluminum [21]. They also decrease the open-circuit potential and corrosion current density and increase the pitting potential of intermetallic Al_2_Cu in 0.5 M NaCl solutions at pH = 9.17 [22]. Decavanadate ions, such as [HV_10_O_28_]^5−^ and [H_2_V_10_O_28_]^4−^, which are stable in mildly and strongly acidic solutions, do not exhibit an inhibiting effect on the 2024 alloy [21,23]. Vanadium isopolyoxoanions, however, were not tested as corrosion inhibitors of intermetallic Al_2_Cu in acidic solutions. The idea of such studies is related to development of new corrosion inhibitors for aluminum alloys in acidic media. Although Al_2_Cu intermetallic in the alloy does not need corrosion protection because it is more noble than the matrix, the situation may change in the presence of the inhibitor. When it is adsorbed onto anodic sites e.g., on the matrix it may change polarity in the corrosion cell and Al_2_Cu phase will become exposed to corrosion. Alternatively, when Al_2_Cu surface is covered with an insoluble film, the cathodic reaction will be hampered, and corrosion rate of the alloy reduced. Anyway, successful replacement of toxic hexavalent chromium species requires systematic studies of the influence of potential corrosion inhibitors on the corrosion behavior of both the alloy and its microstructural constituents, both cathodic and anodic with respect to the matrix.

## 2. Materials and Methods

Al_2_Cu alloy, ca. 4.5 g, was obtained by melting of Al and Cu (99.999 wt.% purity, Alfa Aesar, Lancashire, UK under Ar atmosphere in an electric arc furnace using tungsten electrode. Weight loss of the specimen due to vaporization during melting was negligible (<0.1%). The alloy was annealed in air, *T* = 823 K, *t* = 30 h, cut using an electrical discharge machine, mounted in an epoxy resin, grinded with SiC abrasive paper (320 and 500 grit), rinsed with water and isopropyl alcohol, and air-dried. Geometric surface area of the Al_2_Cu specimen was 0.79 cm^2^.

Microstructure of obtained specimen was revealed using Keller’s reagent (HF 1% *v*/*v*, HCl 1.5% *v*/*v*, HNO_3_ 2.5% *v*/*v*, *t* = 45 s at the room temperature) and examined using light microscope Leica DMI 3000M, Leica Microsystems CMS GmbH, Wetzlar, Germany. Phase composition was confirmed using ARL X’Tra X-ray diffractometer, Thermo Fisher Scientific Inc., Waltham, U.S., with Cu K_α_ radiation source, and STA 449 F3 Jupiter thermal analyzer, Netzsch-Gerätebau GmbH, Selb, Germany. Differential Scanning Calorimetry (DSC) curves were recorded for 42 mg of the alloy, in PtRh20 crucible with Al_2_O_3_ liner obtained from Netzsch-Gerätebau GmbH, under He atmosphere, with heating rate 5 K·min^−1^.

Corrosion rate of Al_2_Cu phase was determined as a function of pH and vanadium initial concentration *c*_V_. Na_3_VO_4_ was dissolved in 0.04 M sulfuric(VI) acid and solutions with *c*_V_ = 0, 10, 50, 100, and 200 mM were obtained. Their pH was adjusted using concentrated H_2_SO_4_ at 1.3 and 2.5. UV-Vis absorption spectra of these solutions were measured using Cary 60 spectrophotometer, Agilent Technologies Inc., Santa Clara, CA, US from 1100 to 190 nm in the quartz cuvettes with 10 mm optical path length. Samples were diluted 300 times using H_2_SO_4_ solutions with pH 1.3 or 2.5.

The volume of solutions used for corrosion tests was 100 cm^3^, temperature was 303 K and immersion time was equal to 576 min. All the solutions were in equilibrium with air. Aluminum and copper concentrations in solutions after corrosion tests were measured using inductively coupled plasma-optical emission spectroscopy ICP-OES (Ultima 2 Horiba Jobin Yvon SAS, Longjumeau, France) and corrosion rates were calculated.

Microscopic examination of the surface of selected corroded Al_2_Cu specimens was performed using Phenom XL scanning electron microscope equipped with energy-dispersive X-ray spectrometer (EDX), Thermo Fisher Scientific Inc., Waltham, U.S. Further examination of corrosion product precipitated on the specimens was performed using X-ray photoelectron spectroscopy. Scanning XPS Microprobe PHI 5000 Versa Probe II (ULVAC-PHI, Chigasaki, Japan) system with microfocused (100 µm, 25 W) Al Kα X-ray beam was applied. The photoelectron spectra were acquired from 400 × 400 µm areas with analyzer pass energy of 46.95 eV and a photoelectron take-off angle of 45°. The charging effect was compensated using a dual-beam charge neutralizer. The operating pressure in the analytical chamber was <5 × 10^−7^ Pa. All XPS peaks were referenced to the oxide O1s peak at 531.0 eV. Pre-sputtering of the samples was performed by 10 min. with an argon gas cluster ion beam (Ar-GCIB) and the average Ar cluster size was 4000 atoms. The beam energy was set to 10 keV, beam current was 5 nA, and the sputtered area was 3 × 3 mm^2^. The samples were rotated during pre-sputtering (Zalar rotation) to minimize surface roughening due to cluster bombardment. Application of the pre-sputtering by Ar-GCIB allowed to remove potential contamination on sample surfaces. Spectrum background was subtracted using the Shirley method. PHI MultiPak^TM^ data analysis software (version 9.9.0.8) was used to calculate elemental compositions from the peak areas.

Electrochemical tests were conducted in a 3-electrode electrochemical cell open to air. Platinum wire with the surface area 20 cm^2^ was applied as a counter electrode and Ag|AgCl (3 M KCl) as a reference electrode. The latter was placed in a Luggin probe filled with 1 M KNO_3_ solution. The solution volume was 100 cm^3^ and temperature 303 K. The electrochemical cell was placed in a Faraday cage and connected to a SP-300 potentiostat, Bio-Logic SAS, Seyssinet-Pariset, France.

**Figure 1:**
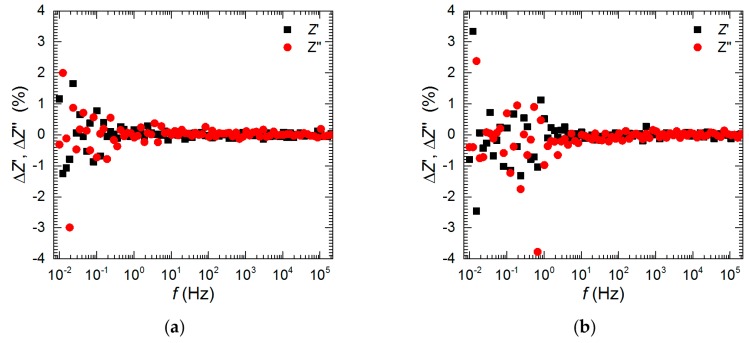
Residual graphs for Kramers–Kronig transformations performed using KK Test software: (**a**) *c*_V_ = 50 mM, (**b**) *c*_V_ = 100 mM, pH = 1.3, *t* = 576 min, *T* = 303 K.

The open-circuit potential (OCP) of Al_2_Cu was recorded for 576 min as a function of vanadium initial concentration, *c*_V_ = 0, 10, 50, 100, and 200 mM, and pH = 1.3 and 2.5. Further electrochemical analysis was limited to the following cases. Impedance spectra of the intermetallic together with polarization curves were measured at pH = 1.3, *t* = 576 min, as a function of vanadium concentration. An additional impedance spectrum was measured at pH = 2.5, *c*_V_ = 200 mM, *t* = 159 min to confirm the possibility of temporary corrosion inhibition at these conditions. The kinetics of corrosion of Al_2_Cu was studied for *c*_V_ = 0 mM at pH = 1.3 and 2.5. In this particular case, the impedance spectra were recorded after *t* = 159, 298, 437, and 576 min. The frequency domain of all the measured impedance spectra was between 200 kHz and 10 mHz. A sinusoidal perturbation of potential equal to 5 mV of a root mean square was applied. The impedance spectra were validated using a Kramers–Kronig transformation with a KK Test software, version 1.01 [24,25]. Examples of obtained results are presented for *c*_V_ = 50 and 100 mM in Figure 1a and b, respectively. Random spread of residuals Z′ and Z″ indicates sufficient stability of the studied system. These residuals were obtained using Equations (1) and (2) [24,25]:

(1) $$\Delta Z^{\prime }=\frac{{Z^{\prime }}_{\mathrm{meas}}-{Z^{\prime }}_{\mathrm{calc}}}{\left|Z_{\mathrm{calc}}\right|}$$

(2) $$\Delta Z^{\prime \prime }=\frac{{Z^{\prime \prime }}_{\mathrm{meas}}-{Z^{\prime \prime }}_{\mathrm{calc}}}{\left|Z_{\mathrm{calc}}\right|}$$

where *Z′*_meas_ and *Z*″_meas_ stand for measured values of a real and imaginary parts of the impedance, for a given frequency, whereas *Z′*_calc_ and *Z*″_calc_ were calculated using KK Test software. $\left|Z_{\mathrm{calc}}\right|$ indicates modulus of the calculated impedance.

After successful validation, impedance spectra were approximated using the appropriate electrical equivalent circuit (EEC) in Zview software (Scribner Associates, version 3.5d). The square of the standard deviation between the measured and calculated data, called in the software as *χ*^2^ (chi squared) and the weighted sum of squares of differences between measured and calculated data *S* were used to estimate the quality of the fit. Values of the fitted parameters were normalized to the geometric surface area of the specimens (0.79 cm^2^).

Polarization curves were measured in the vicinity of the corrosion potential (±25 mV) and fitted using Equation (3) [26]:

(3) $$i=\frac{1}{2.303R_{p}}\frac{\beta_{a}\beta_{c}}{\beta_{a}+\beta_{c}}\left[\exp\left(\frac{2.303\eta }{\beta_{a}}\right)-\exp\left(\frac{-2.303\eta }{\beta_{c}}\right)\right]$$

where *i* is the current density, *R*_p_ the polarization resistance, *η*—overpotential, *β*_a_ and *β*_c_—Tafel constants for the anodic and cathodic process, respectively.

## 3. Results and Discussion

### 3.1. Characterization of Al_2_Cu Intermetallic Phase

**Figure 2:**
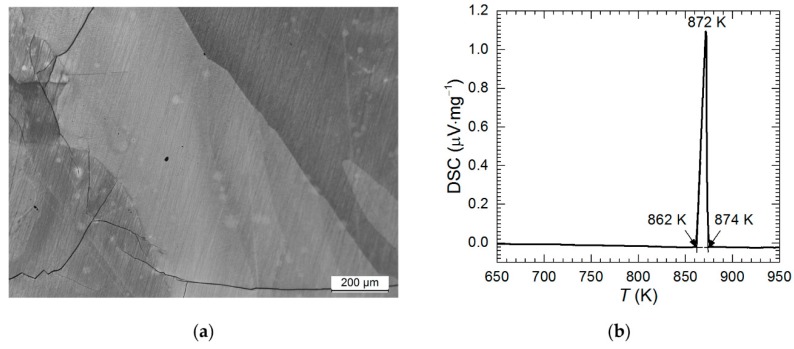
Characterization of phase composition of Al_2_Cu intermetallic phase: (**a**) photomicrograph of the specimen etched using Keller’s reagent; (**b**) DSC curve obtained for heating rate 5 K·min^−1^ in He atmosphere.

In our previous work it was shown that the Al_2_Cu intermetallic was contaminated with a small amount of eutectic mixture of Al_2_Cu and Al(Cu) solid solution [27]. This phase composition, however, is non-equilibrium one due to rapid crystallization. Thus, the single-phase alloy in the present work was obtained after prolonged annealing (30 h) at 550 °C. This was confirmed using X-ray diffraction (XRD) method. All the diffraction lines were ascribed to Al_2_Cu tetragonal phase, International Centre for Diffraction Data (ICDD) card 04-001-0923 (data not shown). Because XRD is not able to detect possible small amount of impurities, additional characterization, using light microscopy (LM) and DSC, was conducted (Figure 2).

Eutectic mixture would appear on the photomicrograph at the grain boundaries of Al_2_Cu hypereutectic crystals. This however was not observed (Figure 2a). LM examination of the metallographic specimen revealed only large grains of Al_2_Cu phase and cracks. The latter arose during crystallization because Al_2_Cu phase is very hard and brittle.

DSC analysis definitely confirmed that the single-phase alloy was obtained. Only one endothermic peak appeared during heating with the onset at 862 K (Figure 2b). This temperature is very close to the peritectic temperature 863.5 K and well above the eutectic temperature (823 K) [28].

### 3.2. Corrosion Rate of Al_2_Cu Intermetallic Phase

**Figure 3:**
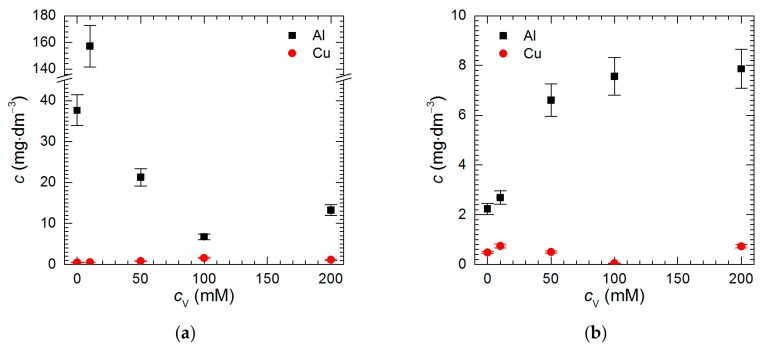
Concentration of Cu and Al in H_2_SO_4_ solutions after *t* = 576 min immersion of intermetallic Al_2_Cu, *T* = 303 K as a function of the initial concentration of Na_3_VO_4_: (**a**) pH = 1.3; (**b**) pH = 2.5.

Isopolyoxovanadates were tested as potential corrosion inhibitors of intermetallic Al_2_Cu in H_2_SO_4_ solutions at pH = 1.3 and 2.5. It was observed that at both pH, Al concentration in the solutions after *t* = 576 min Al_2_Cu exposition was higher than that of Cu (Figure 3a,b). This suggests that the intermetallic corroded selectively. The higher the difference between concentration of both metals, the more severe possible dealloying of the intermetallic. Thus, at pH = 2.5, it occurred to much lesser extent when compared to pH = 1.3.

**Figure 4:**
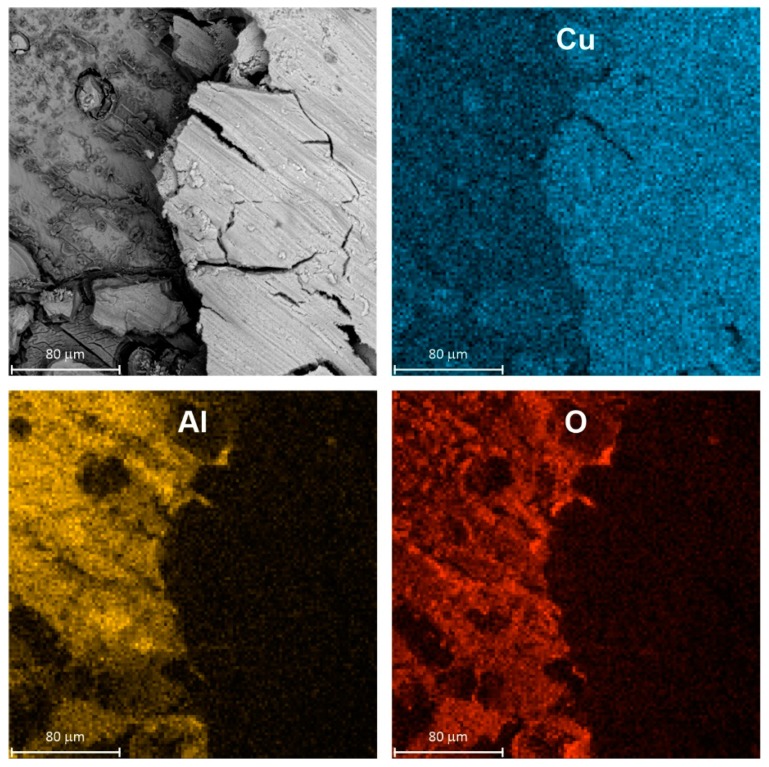
Micrograph of intermetallic Al_2_Cu after corrosion in H_2_SO_4_ solution without Na_3_VO_4_, pH = 1.3, immersion time *t* = 576 min, *T* = 303 K together with spatial distribution of elements, color intensity differences on EDX maps occur due to different concentration of elements and are also related to height of the specimen.

SEM examination of a specimen after corrosion confirmed its selective character. The surface of intermetallic Al_2_Cu became porous and enriched with copper (Figure 4). The porous outer layer can be easily peeled off from the substrate e.g., during specimen rinsing or drying after the corrosion test. It should be noted here that color intensity differences on EDX maps came not only from the spatial distribution of elements but also from differences in height of the specimen.

**Figure 5:**
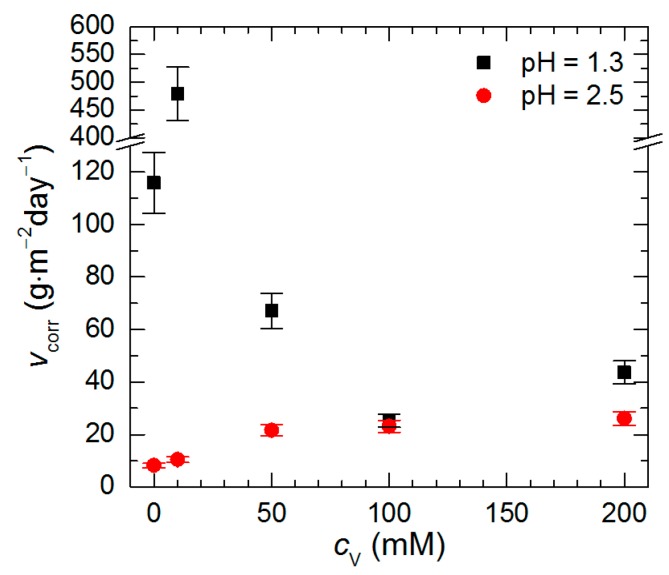
Corrosion rate of intermetallic Al_2_Cu in H_2_SO_4_ solutions after *t* = 576 min at *T* = 303 K as a function of pH and initial concentration of Na_3_VO_4._

Data presented in Figure 3 were used for calculation of Al_2_Cu corrosion rate (Figure 5). At pH = 2.5 its lowest value was obtained for *c*_V_ = 0 mM. It then increased with increasing initial concentration of Na_3_VO_4_, thus, no corrosion inhibition was observed. Although at pH = 1.3, *c*_V_ = 0 mM the corrosion rate of the intermetallic was higher when compared to pH = 2.5, it decreased when *c*_V_ ≥ 50 mM. The lowest values of corrosion rate were obtained at *c*_V_ = 100 and 200 mM. They correspond to inhibition efficiency equal to 78% and 62%, respectively.

**Figure 6:**
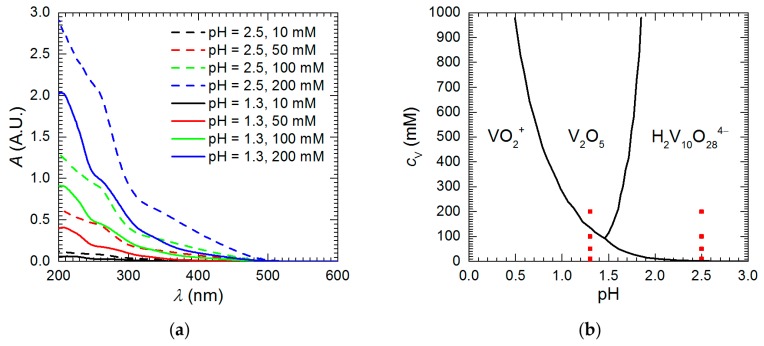
Vanadium speciation in the studied solutions: (**a**) UV-Vis absorption spectra recorded prior corrosion experiments for solutions diluted 300 times; (**b**) approximate distribution diagram of vanadium chemical species in acidic solutions prepared using data from [19], red points indicate chemical composition of solutions studied in this work.

Vanadium speciation in the studied solutions explains differences in the corrosion rate. UV-Vis spectroscopy revealed that different vanadium forms exist at pH = 1.3 and 2.5 (Figure 6a). The shape of the spectra and peak intensities for pH = 2.5 are different when compared to pH = 1.3 at corresponding vanadium initial concentrations. Solutions of isopolyoxovanadates at the higher pH absorb UV and visible light much stronger than at the lower pH. Color difference between vanadium solutions at these two pH values was visible with the unaided eye. It was also observed that change in vanadium initial concentration at a given pH changed only absorption peak intensities, but not their positions. This means that vanadium speciation does not change with increasing *c*_V_ provided that pH is constant. According to thermodynamic considerations, pentavalent vanadium species in strongly acidic solutions exist as pervanadyl VO_2_^+^ or decavanadate H_2_V_10_O_28_^4−^ ions (Figure 6b). Thus, the former is probably responsible for certain extent of corrosion inhibition at pH = 1.3 whereas the latter only accelerated intermetallic corrosion at pH = 2.5.

**Figure 7:**
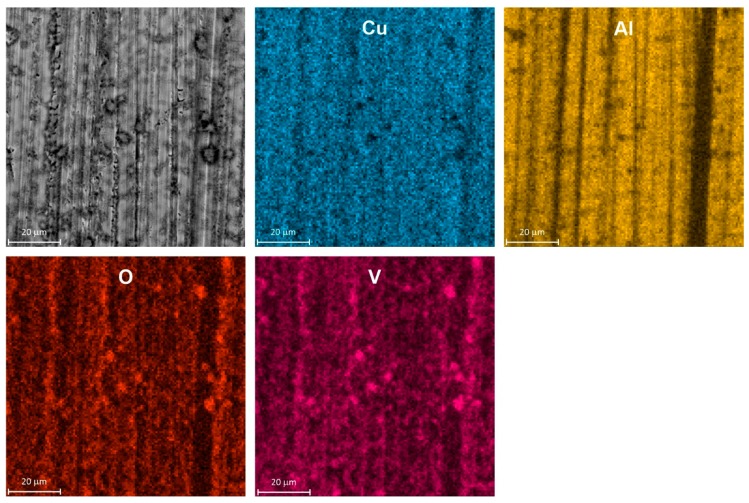
Micrograph of intermetallic Al_2_Cu after corrosion in H_2_SO_4_ solution, *c*_V_ = 100 mM, pH = 1.3, immersion time *t* = 576 min, *T* = 303 K together with spatial distribution of elements, color intensity differences on EDX maps occur due to different concentration of elements and are also related to height of the specimen.

In fact, corrosion inhibition when *c*_V_ ≥ 100 mM was related to precipitation of vanadium-containing film on the surface of the electrode. EDX analysis indicated that Al, Cu, V and O were uniformly distributed on the surface and no severe corrosion loss was observed (Figure 7). Precipitation occurred also within bulk of the solution. According to Figure 6b, V_2_O_5_ should precipitate from the solution at pH = 1.3, when *c*_V_ ≥ 130 mM. This however is only an approximate distribution diagram. Positions of demarcation lines in the diagram depend on the ionic strength of the solution, the counterion as well as temperature. Therefore, precipitation of V_2_O_5_ from solution containing 100 mM of vanadium cannot be excluded.

**Figure 8:**
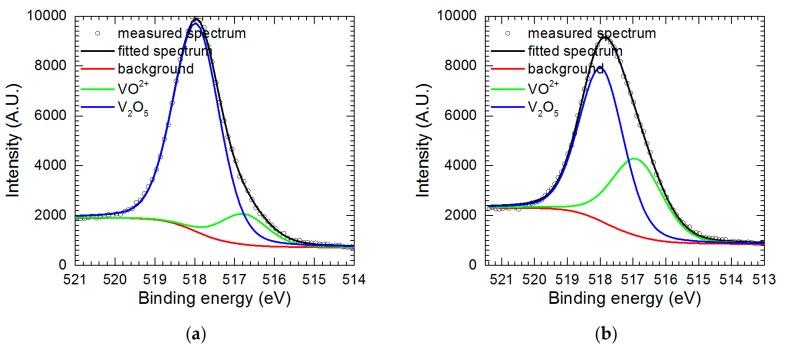
V 2p^3/2^ fitted peaks: (**a**) for powder precipitated from the solution; (**b**) for vanadium-containing film deposited onto electrode.

Further examination of precipitate in the form of powder and thin film on the surface of the electrode was performed using X-ray photoelectron spectroscopy (XPS). Major components of the powder are oxygen and vanadium. Oxygen was mainly bound to vanadium with small admixture of sulfate (data not shown). Much more interesting is V 2p^3/2^ spectrum (Figure 8a). It shows two forms of vanadium at 516.8 eV and 518.0 eV, corresponding to V(IV) and V(V) states, respectively [29]. Therefore, the powder is composed of V_2_O_5_ with a small admixture of adsorbed VO^2+^. Data presented in Table 1 confirms this assumption i.e., the sum of concentrations of oxygen in vanadium pentoxide and vanadyl cation, calculated from the vanadium concentrations, equals to total oxide oxygen concentration.

The spectrum obtained for vanadium-containing film on the electrode surface, besides aforementioned O 1s and V 2p^3/2^ peaks consists also of Al 2s and Cu 2p peaks at 120 and 933 eV, respectively. Al 2s peak overlaps with Cu 3s which occurs around 123 eV. Taking it into consideration Cu 3s peak was eliminated from the Al 2s spectra. Oxygen again is mainly bound to vanadium, with small admixture of sulfate. Vanadium in the film is mainly in the form of V_2_O_5_, but the contribution of VO^2+^ is higher when compared to the powder (Figure 8a, Table 2). It is reasonable because reduction of VO_2_^+^ to VO^2+^ occurs at the electrode surface. Al 2s peak is mainly related to Al^3+^ formed during Al_2_Cu corrosion and adsorbed onto the vanadium-containing film. In the case of copper, it is difficult to determine whether it is in ionic or metallic form. Both possibilities are plausible. Cu is present in the solution due to corrosion process. Its concentration in the solution is ca. 4 times lower when compared to aluminum (Figure 3a). Small contribution from the substrate i.e., Al_2_Cu also cannot be excluded. Big discrepancy between Cu content obtained in two different areas on the specimen seems to justify this speculation. Additional general conclusion is that the chemical composition of the vanadium-containing film is less homogeneous when compared to the powder.

### 3.3. Electrochemical Analysis—Influence of Vanadium Species

**Figure 9:**
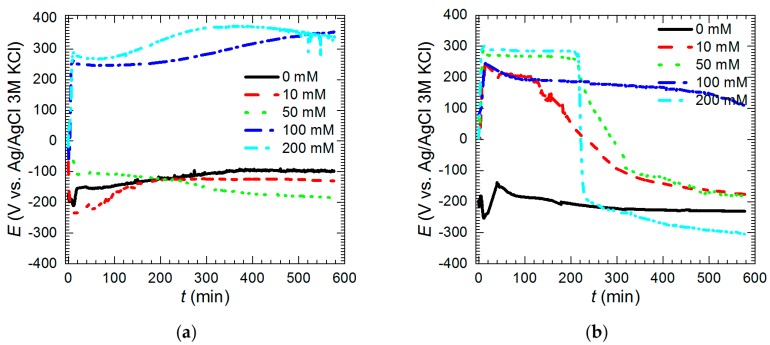
Open-circuit potential of intermetallic Al_2_Cu as a function of time and initial concentration of Na_3_VO_4_ in the solutions: (**a**) pH = 1.3; (**b**) pH = 2.5, *T* = 303 K.

OCP of the intermetallic Al_2_Cu was studied as a function of time and initial concentration of Na_3_VO_4_ in the solutions. When their pH was 1.3 and *c*_V_ = 0 and 10 mM OCP values initially dropped and then slowly increased with time (Figure 9a). When vanadium initial concentration was equal to 50 mM OCP decreased slowly with time. OCP values after *t* = 576 min, for solutions with *c*_V_ ≤ 50 mM at pH = 1.3, were between −99 and −185 mV vs. Ag|AgCl (3 M KCl) electrode. On the contrary, when *c*_V_ = 100 and 200 mM, OCP increased as the time increased and reached 340–360 mV vs. Ag|AgCl (3 M KCl). It should be noted here that equilibrium potential of hydrogen electrode is −287 mV vs. Ag|AgCl (3 M KCl). Thus, one of the possible cathodic process is oxygen reduction. Equilibrium potential of oxygen electrode is 940 mV vs. Ag|AgCl (3 M KCl). Another is reduction of V^V^ to V^IV^ with the equilibrium potential 790 mV vs. Ag|AgCl (3 M KCl) [30]. The latter was confirmed using XPS. Further reduction of tetravalent to trivalent vanadium species also is possible when *c*_V_ ≤ 100 mM, because the equilibrium potential is equal to 127 mV vs. Ag|AgCl (3M KCl) [30]. V^III^ species however were not detected neither in the powder, nor within the vanadium-containing film. When pH = 2.5 OCP values decreased with time (Figure 9b). Interestingly, when *c*_V_ = 50 and 200 mM, plateaus on the OCP vs. time curves were observed. This may indicate temporary effect of corrosion inhibition. OCP values after 576 min for *c*_V_ = 0, 10, 50, and 200 mM were between −150 and −300 vs. Ag|AgCl (3 M KCl). This is slightly lower than pH = 1.3. Selective corrosion in the solutions with pH = 2.5 was less severe when compared to pH = 1.3. Thus, electrodes contained less copper on the surface. This caused lower values of OCP. Relatively high value of OCP was achieved when *c*_V_ = 100 mM. This, however, does not mean lower corrosion rate (Figure 5). At pH = 2.5 the same cathodic processes can occur as mentioned above. Reduction of V^IV^ to V^III^ is possible when *c*_V_ = 10, 50, and 200 mM.

**Figure 10:**
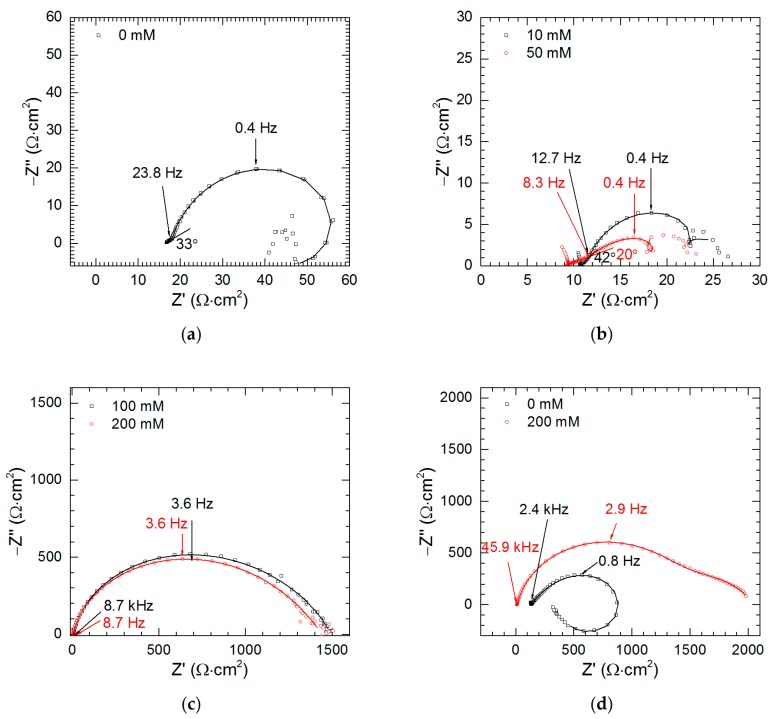
Impedance spectra of intermetallic Al_2_Cu as a function of initial concentration of Na_3_VO_4_ in the solutions: (**a**) 0 mM, pH = 1.3, *t* = 576 min; (**b**) 10 and 50 mM, pH = 1.3, *t* = 576 min; (**c**) 100 and 200 mM, pH = 1.3, *t* = 576 min; (**d**) 0 and 200 mM, pH = 2.5, *t* = 159 min; *T* = 303 K, continuous lines indicate fitted parts of the spectra.

Impedance spectra of intermetallic Al_2_Cu for pH = 1.3, *t* = 576 min were analyzed as a function of sodium orthovanadate initial concentration. This pH was selected because certain degree of corrosion inhibition was achieved on the contrary to pH = 2.5 (Figure 5). The characteristic features of the spectra obtained when *c*_V_ ≤ 50 mM are linear parts at high frequency, *f* ≥ 23.8 Hz (0 mM), 12.7 Hz (10 mM) and 8.3 Hz (50 mM, Figure 10a,b). They have an angle between 20 and 42° with respect to the real axis. This is related to the porous layer formed on the surface due to selective corrosion (Figure 3, Figure 4). The impedance response of the porous electrode was initially introduced by de Levie [31]. According to his model, the Nyquist plot calculated for a single, cylindrical, pore, in the absence of a faradaic current consisted of a straight line with 45° angle at high frequencies and a vertical line at low frequencies. In the real systems, however, it is necessary to include different shape of the pores, size of their openings, possible branching, contribution from the flat part of the electrode on its top and ohmic drop within the solution and electrode material. These factors cause deviation from the theoretical angle or even from the linear shape of the high-frequency part. Due to the charge-transfer processes, semicircle or depressed semicircle appears at lower frequencies instead of the horizontal line [32,33,34]. The high-frequency parts of the spectra are related to geometry of the porous electrode and do not contain valuable information regarding corrosion kinetics. Thus, they were not fitted. Distorted semi-circles observed at medium frequencies (23.8–0.4 Hz for 0 mM, 12.7–0.4 Hz for 10 mM and 8.3–0.4 Hz for 50 mM) correspond to the double-layer capacitance *C*_dl_ in parallel to the charge-transfer resistance *R*_ct_. Due to frequency dispersion, constant phase element CPE was used for fitting instead of the capacitor [32]. Additional one or more time constants can be observed at lower frequencies. They include inductive and capacitive loops and are related to adsorption processes. Scattered impedance values observed at the lowest frequencies were excluded from fitting.

Impedance spectra obtained for *c*_V_ = 100 and 200 mM are different when compared to the other ones. First, linear, high-frequency parts indicating electrode porosity do not exist. Secondly, due to precipitation of vanadium-containing film, much higher impedances were obtained (Figure 10c).

When pH = 2.5, *t* = 159 min, *c*_V_ = 0 and 200 mM also high impedances were obtained (Figure 10d). This confirms temporary corrosion inhibition deduced from OCP vs. time curve (Figure 9b).

**Figure 11:**
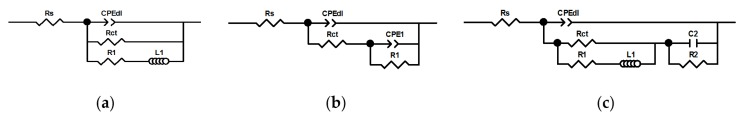
Electrical equivalent circuits used for approximation of the impedance spectra, where *R*_s_ stands for solution resistance, CPE_dl_ is the constant phase element corresponding to double layer capacitance, *R*_ct_ is the charge transfer resistance, other EECs’ elements are related adsorption and precipitation of insoluble film: (**a**) case of selective corrosion, when *c*_V_ = 0 mM, pH = 1.3, *t* ≥ 437 min, *c*_V_ = 0 mM, pH = 2.5; (**b**) case of corrosion inhibition, when when *c*_V_ = 100 and 200 mM, pH = 1.3, *c*_V_ = 200 mM, pH = 2.5, *t* = 159 min; (**c**) case of selective corrosion, when *c*_V_ = 0 mM, pH = 1.3, *t* < 437 min, *c*_V_ = 10 and 50 mM.

Impedance spectra were fitted using EECs presented in Figure 11. Faradaic impedance of the first one (Figure 11a) consists of *R*_ct_, *R*_1_, and L1. This model was initially introduced by Epelboin et al. for passivation of metals. The resistance *R*_1_ was related to changes of the surface coverage or film resistivity with time. The inductance *L*_1_ was inversely proportional to the time constant of this process [35]. In this work, connection of *R*_1_ and *L*_1_ in series is related to adsorption of species formed during the anodic dissolution of the intermetallic Al_2_Cu. These are probably aluminum ions. It should be noted here that detection of different intermediates adsorbed during aluminum corrosion in acids is impossible using EIS [36]. However, in other environments, such as aqueous alkaline and non-aqueous solutions, three step mechanisms of Al dissolution were successfully investigated using EIS [36,37].

The alternative EEC, where passive behavior is modelled using capacitor in parallel to resistor, was also proposed [35]. This model was used in this work for fitting the spectra obtained for *c*_V_ = 100 and 200 mM at pH = 1.3 and *c*_V_ = 200 mM at pH = 2.5, where inductive behavior was not observed (Figure 11b). However, constant phase element CPE_1_ was used instead of the capacitor. *R*_1_ and CPE_1_ depend both on the charge-transfer resistance and faradaic current change caused by increase of the surface coverage, presumably with vanadium-containing film [35].

When *c*_V_ = 10 and 50 mM more complicated equivalent circuit, with additional time constant, was used (Figure 11c). Cao derived general equation of faradaic impedance for electrode processes whose rate depend on electrode potential and two other, potential-dependent, variables such as surface coverage [38]. He also proposed several equivalent circuits applicable for such a case. In this work, these two potential-dependent variables are related to adsorption of two different species, presumably aluminum ions and vanadium isopolyoxoanions. Physical interpretation of inductor *L*_1_, capacitor *C*_2_, and resistors *R*_1_ and *R*_2_, applied in this model, is not straightforward. In general, these parameters are mutually related e.g., *C*_2_ depends on *R*_1_, *R*_2_, *R*_ct_, and *L*_1_. They depend on changes in faradaic current caused by changes in surface coverage and changes in adsorption kinetics caused by electrode potential. In addition, all these parameters depend on both adsorbing species [38].

The results of the impedance spectra fitting are given in Table 3, Table 4 and Table 5.

**Figure 12:**
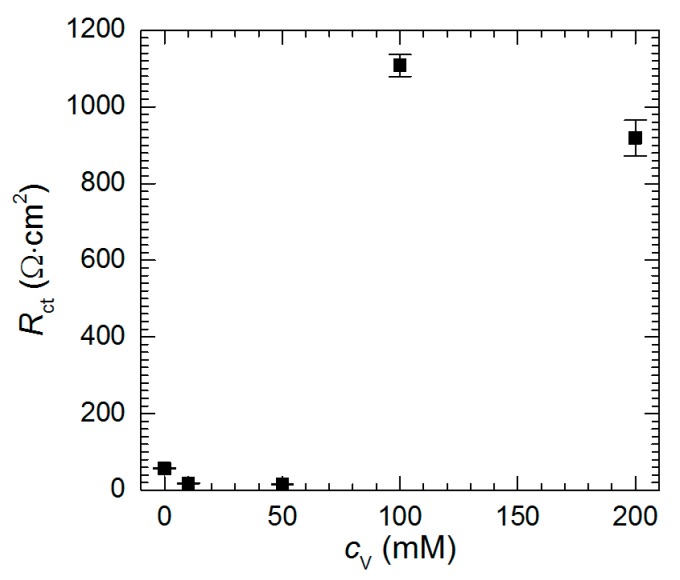
Charge-transfer resistance as a function of initial concentration of sodium orthovanadate in H_2_SO_4_ solution, pH = 1.3, *t* = 576 min, *T* = 303 K.

In general, to estimate the corrosion rate from the impedance spectra one should determine polarization resistance *R*_p_ value i.e., the resistance when *f*→0. Unfortunately, resistances obtained at low frequencies are scattered and reliable values of *R*_p_ cannot be obtained. Approximation of the impedance spectra allowed, however, determination of the charge-transfer resistance. It decreases as *c*_V_ increases from 0 to 50 mM (Figure 12). This indicates that at pH = 1.3 vanadium species, presumably pervanadyl ions, act as additional depolarizers in the corrosion cell. In fact, reduction of pervanadyl to vanadyl was even confirmed for *c*_V_ = 100 mM using XPS. From Figure 5 it can be concluded that certain corrosion inhibition is expected at *c*_V_ = 50 mM. It should be noted here, however, that corrosion rates presented in Figure 5 are averaged over *t* = 576 min whereas impedance spectrum gives information at the moment when it is recorded. In addition, *R*_ct_ values are normalized to geometric surface area of the specimen but not the real one. The latter remains unknown but possibly very high due to intensive selective corrosion [27]. The increase of the real surface area also causes the decrease of the resistance.

When *c*_V_ ≥ 100 mM, *R*_ct_ achieves much higher values. Vanadium-containing film precipitating on the surface of the intermetallic, hampers the charge transfer through the solid/liquid interphase.

The increase of the surface area of the electrode caused by selective corrosion can be easily monitored using the double-layer capacitance. The capacitive behavior of the electrical double layer is usually modelled using CPE instead of capacitor due to frequency dispersion. The impedance of the CPE is given by Equation (4) [32]:

(4) $$Z_{\mathrm{CPE}}=\frac{1}{T_{\mathrm{dl}}(j\omega {)}^{\alpha }},$$

where *T*_dl_ is equal to capacitance when *α* = 1, *ω* is the angular frequency and *j* is imaginary number.

Provided that time constants for the faradaic process are distributed along the surface of the electrode, the double-layer capacitance can be obtained as follows (Equation (5)) [32]:

(5) $$C_{\mathrm{dl}}={T_{\mathrm{dl}}}^{\frac{1}{\alpha }}({\frac{1}{R_{s}}+\frac{1}{R_{\mathrm{ct}}})}^{1-\frac{1}{\alpha }}$$

This equation works well for relatively high *α* values, but when α < 0.85, calculation of *C*_dl_ can lead to significant error [39]. Thus, double-layer capacitance was estimated only for *c*_V_ = 0 mM at pH = 1.3. After *t* = 576 min it is equal to 9.632 (0.482) mF·cm^−2^. Again, it should be noted that capacitance was normalized to geometric surface area of the specimen. The value obtained is 482 times higher when compared to theoretical value 20 µF·cm^−2^ [34]. This is characteristic for highly porous electrodes and is related to their enormous surface area [12,27,33,34].

It should be noted here that resistances *R*_1_ obtained from impedance spectra approximation were very low. Thus, in Table 3 0 Ω values are reported. This is probably caused by a very small diameter of the closed loops e.g., in Figure 10b. Inductances gradually decrease as the surface area of the electrode increases.

Polarization resistances *R*_p_ were estimated using polarization curves recorded in the vicinity of the corrosion potential at pH = 1.3 (data not shown). They were approximated using Equation (3). It can be concluded that *R*_p_ = 32, 16, 15, 912 and 1146 Ω·cm^2^ for *c*_V_ = 0, 10, 50, 100, and 200 mM. Again, the discrepancy between the corrosion rate and polarization resistance exists for *c*_V_ = 50 mM. The possible explanation was given during discussion of *R*_ct_ values. These *R*_p_ values are normalized to geometric surface area of the electrode i.e., they decrease as the surface area increases. The values of Tafel constant were in most cases obtained with very high uncertainties. Furthermore, their practical importance is in this case insignificant, because they characterize all cathodic and anodic processes occurring at the electrode. These two obstacles make calculation of the corrosion current density from polarization resistance impossible.

### 3.4. Electrochemical Analysis—The Kinetics of Selective Corrosion

**Figure 13:**
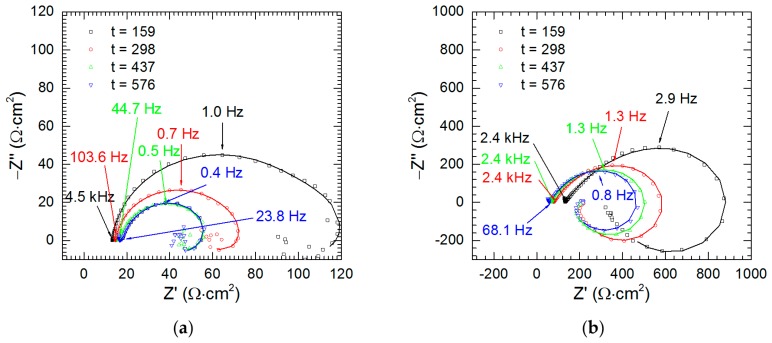
Impedance spectra of intermetallic Al_2_Cu as a function of immersion time: (**a**) pH = 1.3; (**b**) pH = 2.5; *T* = 303 K, *c*_V_ = 0 mM.

The kinetics of corrosion was studied in the solutions without vanadium species at pH = 1.3 and 2.5. The impedance of the intermetallic decreases as the immersion time increases. It is also much lower at pH = 1.3 than at pH = 2.5 (Figure 13). The results of fitting of these spectra are given in Table 5.

**Figure 14:**
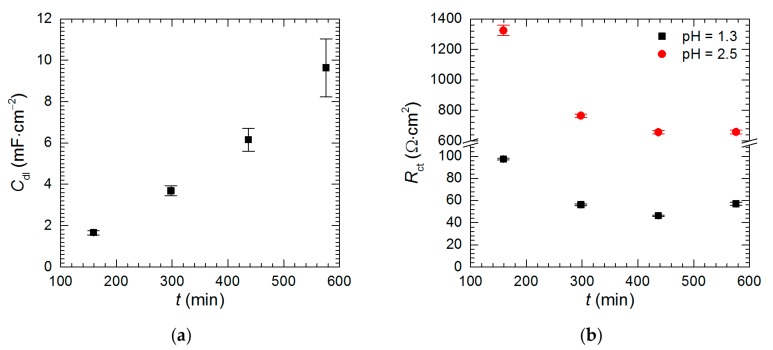
Kinetics of selective corrosion of intermetallic Al_2_Cu: (**a**) double-layer capacitance at pH = 1.3; (**b**) charge-transfer resistance at pH = 1.3 and= 2.5; *c*_V_ = 0 mM, *T* = 303 K.

Intermetallic Al_2_Cu corrodes selectively in acidic solutions. This process is more severe at pH = 1.3 than 2.5 (Figure 3). In the former case double-layer capacitance was calculated using Equation (5). Obtained values are high and increase with time due to increase of the surface area of the electrode (Figure 14a). In the latter case *C*_dl_ was not calculated because *α*_dl_ < 0.85. Charge-transfer resistance, in turn, was determined for both cases (Figure 14b). It decreases with time as already explained. Approximately 10-fold lower values were obtained for pH = 1.3.

**Figure 15:**
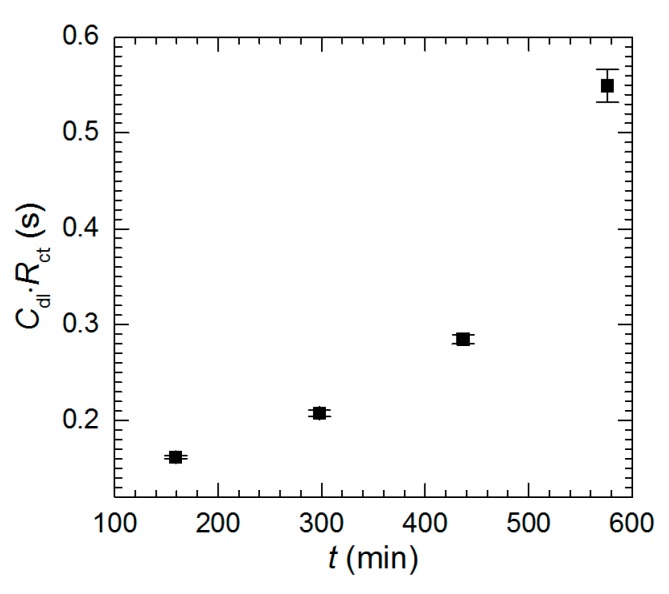
*C*_dl_·*R*_ct_ product as a function of immersion time, pH = 1.3, *c*_V_ = 0 mM, *T* = 303 K.

It can be observed that the product *C*_dl_·*R*_ct_, should not depend on the immersion time of the intermetallic. Interestingly, this is not valid at pH = 1.3 (Figure 15). It means that *C*_dl_ and *R*_ct_ changes with time are caused not only by the increases of electrode surface area but also by the change in mechanism. In fact, it is quite reasonable because the chemical composition of the electrode changes with time i.e., aluminum content on the surface decreases as the immersion time increases. The chemical composition of the solution also changes. Its pH raises in the vicinity of the electrode due to oxygen reduction and aluminum concentration increases. The surface of the electrode becomes highly porous and enriched with copper. Copper corrodes in aerated acidic solution when is not electrically coupled with aluminum. At the same time, porous copper structure can locally detach from the surface and dissolve in the solution. Thus, copper ions may easily act as the additional depolarizers in the corrosion cell. These two factors i.e., changes of chemical composition of the electrode and solution probably affect the double-layer capacitance and charge-transfer kinetics.

## 4. Conclusions

Pentavalent vanadium species were tested as corrosion inhibitors of intermetallic Al_2_Cu in H_2_SO_4_ at pH = 1.3 and 2.5, respectively. When pH = 1.3 and *c*_V_ was < 100 mM severe dealloying of the Al_2_Cu phase occurred. The inhibition effect, observed when *c*_V_ was ≥ 100 mM, was mainly related to precipitation of vanadium-containing film on the surface of the electrode and in the bulk of the solution. XPS spectra analysis showed that V_2_O_5_ is the main component of the film which agrees with thermodynamic considerations. Precipitation of vanadium pentoxide within the bulk of the solution makes application of vanadium species, possibly pervanadyl ions, as corrosion inhibitors of Al_2_Cu at pH = 1.3 impractical. However, their influence on the corrosion behavior of an Al-Cu solid solution and other intermetallics, existing in aluminum alloys, in H_2_SO_4_ should also be studied. When pH = 2.5 and vanadium was probably in the form of decavanadate ions, corrosion of the intermetallic was not inhibited. On the contrary, it was accelerated due to introduction of additional depolarizer to the corrosion cell. At the same time, corrosion rate was lower when compared to pH = 1.3. The corrosion mechanism of Al_2_Cu is complex. Several cathodic processes are possible in the studied system. In addition, surface area of the electrode changes with time which complicates the comparison between the impedance spectra and impede calculation of corrosion current density.

## Figures and Tables

**Table 1:** The contribution of fitted peaks for powder sample (at.%).

Measurement	O 1s	V 2p	
	O^2−^	V^5+^	V^4+^
1	69.8	26.3	3.9
2	68.5	26.3	5.2
Average	69.2	26.3	4.6

**Table 2:** The contribution of fitted peaks for vanadium-containing film on the Al_2_Cu (at.%).

Measurement	O 1s	V 2p		A l2s	Cu 2p
	O^2−^	V^5+^	V^4+^	Al^3+^	Cu^2+^/Cu
1	54.8	16.0	9.1	19.0	1.2
2	61.9	15.7	6.9	15.3	0.3
Average	58.3	15.8	8.0	17.1	0.7

**Table 3:** The results of fitting of the impedance spectra obtained at pH = 1.3 using EEC from Figure 11a,c: χ^2^ and *S* indicate the quality of the fit (chi-square and residual sum of squares, respectively); *R*_s_ is the solution resistance; *T*_dl_ is the parameter of the constant phase element CPE_dl_ representing the electrical double layer; α_dl_ is the exponent of the CPE; *R*_ct_ is the charge-transfer resistance; *L*_1_, *R*_1_, *R*_2_, and *C*_2_ are the inductance, resistances, and capacitance related to the adsorption processes, respectively, uncertainties of the fitted parameters are given in brackets.

Experimental Conditions	χ^2^	S	*R*_s_ (Ω·cm^2^)	*T*_dl_ (mF·s^α−1^·cm^−2^)	α_dl_	*R*_ct_ (Ω·cm^2^)	*R*_1_ (Ω·cm^2^)	*L*_1_ (H·cm^2^)	*C*_2_ (mF·cm^−2^)	*R*_2_ (Ω·cm^2^)
*cV* = 0 mM, *t* = 159 min	1.05 × 10^−4^	0.11	13 (1)	2.055 (0.022)	0.95 (0.01)	97 (1)	0	211 (16)	17.805 (1.368)	91 (2)
*cV* = 0 mM, *t* = 298 min	5.46 × 10^−4^	0.04	15 (1)	4.201 (0.067)	0.96 (0.01)	56 (1)	0	95 (6)	28.958 (2.534)	44 (1)
*cV* = 0 mM, *t* = 437 min	2.23 × 10^−4^	0.01	17 (1)	7.804 (0.083)	0.91 (0.01)	46 (1)	61 (1)	121 (4)		
*cV* = 0 mM, *t* = 576 min	1.59 × 10^−4^	0.01	18 (1)	12.856 (0.168)	0.86 (0.01)	57 (1)	36 (1)	65 (2)		
*cV* = 10 mM, *t* = 576 min	3.76 × 10^−4^	0.02	11 (1)	35.294 (1.202)	0.85 (0.02)	18 (1)	0	17 (1)	99.884 (6.842)	16 (1)
*cV* = 50 mM, *t* = 576 min	2.59 × 10^−4^	0.01	11 (1)	66.191 (2.919)	0.60 (0.03)	15 (1)	0	23 (1)	201.722 (17.043)	17 (1)

**Table 4:** The results of fitting of the impedance spectra obtained when corrosion was inhibited, using EEC from Figure 11b: χ^2^ and *S* indicate the quality of the fit (chi-square and residual sum of squares, respectively); *R*_s_ is the solution resistance; *T*_dl_ is the parameter of the constant phase element CPE_dl_ representing the electrical double layer; α_dl_ is the exponent of the CPE; *R*ct is the charge-transfer resistance; *R*_1_, *T*_1_ and *α*_1_ are the resistance and constant phase element CPE_1_ parameters related to the adsorption/formation of a protective layer processes, respectively, uncertainties of the fitted parameters are given in brackets.

Experimental Conditions		χ^2^	S	*R*_s_ (Ω·cm^2^)	*T*_dl_ (mF·s^α^−1^^·cm^−2^)	α_dl_	*R*_ct_ (Ω·cm^2^)	*R*_1_ (Ω·cm^2^)	*T*_1_ (mF·s^α−1^·cm^−2^)	α_1_
pH = 1.3	*cV* = 100 mM, *t* = 576 min	2.68 × 10^−4^	0.03	7 (1)	0.043 (0.001)	0.88 (0.01)	1108 (29)	379 (33)	0.660 (0.083)	0.64 (0.04)
	*cV* = 200 mM, *t* = 576 min	2.42 × 10^−4^	0.03	5 (1)	0.044 (0.001)	0.88 (0.01)	919 (47)	521 (52)	0.437 (0.066)	0.54 (0.03)
pH = 2.5	*cV* = 200 mM, *t* = 159 min	2.91 × 10^−4^	0.04	11 (1)	0.048 (0.001)	0.87 (0.01)	1450 (11)	581 (21)	2.205 (0.117)	0.68 (0.02)

**Table 5:** The results of fitting of the impedance spectra obtained at pH = 2.5 using EEC from Figure 11a: χ^2^ and *S* indicate the quality of the fit (chi-square and residual sum of squares, respectively); *R*_s_ is the solution resistance; *T*_dl_ is the constant phase element CPE_dl_ parameter representing the electrical double layer; α_dl_ is the exponent of the CPE; *R*_ct_ is the charge-transfer resistance; *L*_1_ and *R*_1_ are the inductance and resistance related to the adsorption processes, respectively, uncertainties of the fitted parameters are given in brackets.

Experimental Conditions	χ^2^	S	*R*_s_ (Ω·cm^2^)	*T*_dl_ (mF·s^α−1^·cm^−2^)	α_dl_	*R*_ct_ (Ω·cm^2^)	*R*_1_ (Ω·cm^2^)	*L*_1_ (H·cm^2^)
*cV* = 0 mM, *t* = 159 min	7.13 × 10^^−4^^	0.07	133 (1)	0.557 (0.009)	0.64 (0.01)	1325 (33)	294 (6)	772 (11)
*cV* = 0 mM, *t* = 298 min	4.49 × 10^^−4^^	0.04	80 (1)	0.587 (0.008)	0.68 (0.01)	766 (11)	150 (3)	398 (4)
*cV* = 0 mM, *t* = 437 min	8.39 × 10^^−4^^	0.08	65 (1)	0.722 (0.011)	0.70 (0.01)	657 (12)	133 (2)	368 (4)
*cV* = 0 mM, *t* = 576 min	1.97 × 10^^−4^^	0.01	61 (1)	0.966 (0.012)	0.71 (0.01)	658 (12)	138 (2)	356 (3)
